# Characterisation of Tobacco Use and its Associated Factors Among Older Youths in an Urban Setting: The Case of Wakiso, Uganda

**DOI:** 10.24248/eahrj.v8i2.788

**Published:** 2024-06-26

**Authors:** Alex Daama, Stevens Kisaka, Stephen Mugamba, Emmanuel Kyasanku, Grace Kigozi Nalwoga, Asani Kasango, Robert Bulamba, James Menya Nkale, Fred Nalugoda, Gertrude Nakigozi, Godfrey Kigozi, Rawlance Ndejjo, Joseph Kagaayi

**Affiliations:** a Africa Medical & Behavioral Sciences Organization, Kampala, Uganda; b Makerere University College of Health Sciences, Kampala, Uganda

## Abstract

**Introduction:**

Tobacco smoking increases the risk of death from many diseases, including ischemic heart disease, cancer, stroke, chronic obstructive pulmonary disease, diabetes, and other fatal and non-fatal diseases. Efforts have been invested towards cessation of tobacco smoking among youths aged 18-35 years. However, population-based data is limited on tobacco smoking in Wakiso district among youths. Therefore, this study aimed to determine the prevalence and factors associated with tobacco smoking among youths aged 18-35 years in Wakiso district Uganda.

**Methods:**

Data from a Population-based survey in Wakiso district collected between October 2019 and September 2020 were used to determine the prevalence of tobacco smoking and associated factors. A cross-sectional design was employed. This study used multivariable logistic regression to estimate odds ratios and 95% confidence intervals (CI) for the association between tobacco smoking and various factors among youths aged (18-35) years in Wakiso district Uganda.

**Results:**

A total of 1,092 participants were enrolled of whom 631(57.8%) were females. The mean age was 25.8 (SD=4.8) years. A total of 35 (3.2 %) reported current tobacco smoking while 64(5.9%) ever used tobacco. The mean age at smoking initiation was 20.6 (SD= 5.3) years. In the multivariable analysis, age groups 25-29 years (aOR= 3.66, [95% CI: 1.15, 11.65]) and 30-35 years (aOR= 4.26, [95% CI; 1.32, 13.72]) were more likely to smoke compared to those under 25 years). Other positively associated factors included alcohol users (aOR= 4.86, [95% CI: 2.01, 11.74]), HIV positive status (aOR= 5.43, [95% CI: 1.86, 15.86]), living with friends or relatives who smoke (aOR=9.93, [95% CI: 1.86, 15.86]), and being male (aOR=4.50 [95% CI; 1.82, 11.13]).

**Conclusion:**

Overall tobacco smoking among youths aged 18-35 years is low compared to national prevalence of 9%. However, the focus should be on males, older youths, alcohol users, and HIV-positive youths including those living with friends or relatives who smoke.

## INTRODUCTION

Globally, much as more efforts are focused on cessation of tobacco smoking following recommendations from research,^1–3^ it remains a growing public health concern especially among youths in developing countries.^4,5^

Tobacco smoking is responsible for over 8 million deaths annually with seven million deaths directly due to tobacco smoking, while 1.2 million are a result of exposure to secondhand smoke.^6^ A tobacco smoker dies 10 years earlier and starts to suffer from disability 12 years earlier than the general population.^7^

Among the 8 million deaths linked to tobacco smoking, 80% take place in low and middle-income countries (LMICs).^8^ While Sub-Saharan Africa boasts one of the lowest tobacco use prevalence rates and has a relatively youthful population, the region has experienced the most significant relative surge in the number of tobacco users compared to other regions.^9^ This trend positions Sub-Saharan Africa as the anticipated future epicenter of the tobacco epidemic.^10^ World Health Organization estimated that approximately 10% of Uganda's population, equivalent to around 1.8 million people, were smokers in 2016.^11^ However, comprehensive data on smoking in the general population is limited. A nationwide survey conducted in 2014 revealed that 7.4% of participants were daily smokers, with 79.3% of them being males. The highest smoking rate was observed in individuals aged 30–49 years.^12^

The World Health Organization (WHO) Global Status report on Non-Communicable Diseases (NCDs) indicated that in Uganda, tobacco smoking was a major risk factor for NCDs which account for 25 % of all deaths in the country.^13^ Unfortunately, current information to characterize tobacco smoking and associated factors in Uganda is limited. Uganda is one of the countries with the largest population of young people which affects all the health indicators.^14^ This observation calls for improved strategies for tobacco control in these regions.^6^ Smoking during this age category (15-30 years) tends to increase the risk of addiction,^15^ and hence this puts them on more regular smoking. ^16^ A nationwide study by Kabwama et al conducted seven years ago (between March-July, 2014) among persons aged 18-69 years, revealed that the prevalence of smoking was 9.2%.^12^

Characterization of tobacco use is important in urban setting because most youths are vulnerable to smoking given that majority are unemployed, poorest and disadvantaged populations and live in urban areas more especially slums hence increasing the risk of NCDs. There are some studies that have characterized tobacco use in South Africa, Egypt, Nigeria and Bangladesh. These have indicated that personal and behavioral risks are related to adolescents' smoking. ^17–20^ However, in Ugandan communities, this information is scarce and not readily available.

Efforts to curb tobacco smoking, led by both governmental and non-governmental organizations, encompass a range of interventions such as bans on advertising, promotion, and sponsorship, smoke-free policies, adjustments to tobacco prices and taxes, as well as health warnings and education initiatives. ^21^ Additionally, various community-based organizations have played a role in mitigating tobacco use and substance abuse among youths.^22^ These community efforts involve diverse activities like debates addressing alcohol abuse and tobacco use, poetry sessions highlighting the risks of tobacco smoking and alcohol consumption, and engaging in sports activities such as netball and football. Skits depicting the dangers of alcohol and tobacco use are also utilized, creating a fun yet informative platform for sensitizing youths about the hazards associated with these substances. The overarching message, encapsulated in the slogan “Say No to Alcohol, drugs, and tobacco,” aims to foster a healthy body and society.

Despite these commendable efforts, there is a gap in documenting the prevalence and factors associated with tobacco smoking among youths. Therefore, this study sought to address this gap by investigating the prevalence and associated factors in rural, peri-urban, and urban areas of Wakiso district, with the ultimate goal of formulating targeted recommendations to address this behavior effectively.

## METHODS

### Study Setting

This study used information collected from Africa Medical and Behavioral Sciences Organization (AMBSO) a Population Health Surveillance (APHS) community cohort study in Wakiso district. APHS cohort started in 2018 and is an open population-based cohort which enrolls about 5,000 consenting persons aged 13 years and above in three communities stratified into rural, urban, and semi-urban areas distributed in Wakiso district. ^23^ APHS operates in two Ugandan districts: Wakiso, which surrounds the capital city of Kampala, located in Uganda's Central region; and Hoima in the Western region, 200 km northwest of Kampala. In both regions, urban, semi-urban and rural communities are surveyed. At the initial census, areas were selected through a community mapping exercise comparing the population structure of areas with different distances from urban centers and varying degrees of cultivation. The aim was to obtain a representative sample of households typical for the different community types (rural, semi-urban, urban) and with varying socioeconomic status. ^24^

These communities include Sentema, Lukwanga and Kazo. Sentema is in Wakiso District,. Kazo is one of the most densely populated slums of Nansana municipality with a population of more than 5,000 residents, it is just 5 km from Kampala city Centre in Uganda. From a laid-back suburb on the fringes of Kampala just ten years ago, the area is now teeming with commercial and residential properties, occupying nearly every inch.

In most of these communities, more especially Kazo, there are over 100 lodges and 200 open bars, these are always open every day for 24 hours. Some of these sites also act as social hub for singers and these are always accompanied by young people, female sex workers and fisher folks who smoke and use alcohol. ^25^ Residents living in these three communities were randomly selected representing rural, urban and semi-urban sub-counties. Surveys were conducted every 8-12 months. These three communities were purposively selected due to presence of key and priority populations especially in the slum areas of the division proximal to Kampala city.

The APHS conducted household census to enumerate eligible participants, obtaining demographic information such as sex, age, relationship to the household head, marital status, residence status among others. The APHS aims have been described. ^23,24,26^ In brief the population based cohort is aimed at generating empirical data to inform policy regarding health status of the population by ensuring disease trends and health determinants are monitored over time. Consenting individuals are interviewed by same sex interviewers to collect annual actual socio-demographic characteristics, sexual behavior, reproductive health, communicable (STIs including HIV) and non-communicable diseases (cardiovascular diseases, cancers), nutritional status, risky health behaviors (Alcohol use, illicit drug use, and tobacco smoking), male circumcision, mental health among others with over 1,000 consenting youths.

The APHS survey recruited 1,092 participants out of 3,166 persons who were eligible and targeted for survey. Two weeks to one month prior to the survey team, all community members both participants and non-participants were mobilized through community structures, phone calls and home visits to attend community health education meetings.

### Type and Period

This was a cross sectional study that utilized secondary data collected between November 2019 and October 2020.

### Study Population

Youths aged 18-35 years in the APHSin urban, rural and semi-urban areas of Wakiso district, who responded to tobacco smoking modules in the semi structured questionnaire administered between November 2019 and October 2020. The study population comprised of all consenting individuals aged (18-35) years of the cohort. The smoking question (history of smoking, current smoking status, age at initiation of smoking, period of smoking and so on).

### Inclusion and Non-inclusion Criteria

Participants were included in the study if they were aged 18-35 years, understood Luganda or English and consented to participate in the study.

### Sampling Techniques:

This study used secondary data from a population-based health survey. The APHS sampling procedure has been previously described ^23,24,26^ and this was also similar to other Population based surveys conducted elsewhere. ^27^ However, briefly the sampling frame considered all households in each selected study community. In each household, all youths aged (18–35) years were considered potential participants. Different communities were chosen using a multistage sampling technique. The population composition of locations with varying distances from urban centers and agriculture was compared, and community mapping was used to choose the research sites for APHS. ^24^ A representative sample of homes from the various communities was to be chosen as the main goal. Each household's possible participants for the annual survey were all individuals who were at least 13 years old.

### Data Collection

The APHS conducted household census to enumerate eligible participants, obtaining demographic information such as sex, age, relationship to the household head, marital status, residence status among others. Following enumeration of eligible households, census forms were double entered by experienced data entrants and survey activities were performed. Two weeks to one month prior to survey team, all community members both participants and non-participants were mobilized through community structures, phone calls and home visits to attend community health education meetings. During survey activities, consenting individuals are interviewed by same sex interviewers to collect annual actual socio-demographic characteristics, sexual behavior, reproductive health, communicable (STIs including HIV) and NCDs (cardiovascular diseases, cancers), nutritional status, risky health behaviors (Alcohol use, illicit drug use, tobacco smoking & smoking), male circumcision, mental health among others.

### Data Analysis

Descriptive and univariable analysis were performed to describe how the variables are distributed amongst the participants. The frequency distributions for categorical variables were computed. For the continuous variables, we analyzed them preliminarily by use of means (with the standard deviation), modes and median (with the range) using STATA version 14.0.

Prevalence of tobacco smoking was determined through cross-tabulation of tobacco smoking status. Bivariate analysis was conducted between each independent factor against tobacco smoking status and other covariates to determine existing relationships, respectively. Those variables known to be associated with smoking with a *P*< 0.2 as well as those which are biologically plausible, based on existing literature were included in the multivariable model.

Multivariable logistic regression analysis was conducted to estimate odds ratios and 95% confidence intervals for the association between factors and tobacco smoking.

### Ethical Considerations

The APHS survey was approved by a local IRB (International Health Sciences University)-CIU/REC0059, Uganda National Council of Science and Technology (UNCST)-SS4468. Approval for the secondary data analysis was sought from Makerere University School of Public Health Higher Degrees Research and Ethics Committee (HDREC). The data used for analysis had a unique alpha-numeric computer-generated numbers used for participant identification. Therefore, no participant's name nor physical location were reflected in the study data set.

## RESULTS

### Baseline Characteristics of Participants

This study included 1,092 participants whose mean age was 25.9 (SD±4.8) years and majority (447 [40.9%]) were between 18-24 years. More than half of the participants (631 [57.8%]) were females; Majority (618 [56.6%]) were married; (315 [28.9%]) had never been married while (159 [14.6%]) were divorced/separated.

Most participants (629 [57.6%]) had post-primary education while, (431 [39.5%]) had primary level, and only (32 [2.9%]) had no education. Majority of the participants were Christians (775 [71.0%]), Muslims (310 [28.4%]) and (7 [0.6%]) had no religious affiliation. Occupation groups included traders (318 [29.1%]); housework; (242 [22.2%]), agriculture (126 [11.5%]); construction & mechanics workers (120 [11.0%]), Bodaboda men (60 [5.49%]) and other had other occupations (175 [16.0%]). About (74 [6.8%]) of participants were HIV-positive and (753 [69.0%]) drank alcohol (Table 1)

**Table 1.** Baseline Characteristics of Respondents

Sociodemographic characteristics	n	Proportion (%)
Sex		
Male	461	42.2
Female	631	57.8
Age group		
18-24	447	40.9
25-29	360	33.0
30-35	285	26.1
Education level		
None	32	2.9
Primary	431	39.5
Post primary	629	57.6
Occupation		
Agriculturists	126	11.5
Trader	318	29.1
House workers	242	22.2
Construction and mechanics workers	120	11.0
Students	51	4.7
Boda-boda	60	5.5
Other	175	16.0
Marital status		
Married	618	56.6
Currently not married	159	14.6
Never been married	315	28.8
Religion		
None	7	0.6
Christians	775	71.0
Muslims	310	28.4
Alcohol use		
Users	339	31.0
Non-users	753	69.0
HIV Status		
Negative	1,018	93.2
Positive	74	6.8

### Prevalence of Tobacco Smoking Among Youths Aged 18 to 35 Years

Out of 1,092 youths aged 18-35 years, 35 (3.2%) self-reported as current smokers while 64 (5.9%) reported to have ever smoked (Table 2). Therefore, youths who had ever smoked were slightly higher than current smokers. These findings also revealed that the average age at smoking initiation was approximately 20.6 years. The proportion of current smokers versus ever smokers for both males and females were 5.4% (25/461), 1.6% (10/631) versus 8.7% (40/461), 3.8% (24/631), respectively. This difference was statistically significant (*P*<.0001).

**Table 2.** Prevalence of Tobacco Smoking Among Youths aged 18-35 years, N=1,092

Variable	Prevalence of current Smokers n (%) n= 35 (3.2)	Current nonsmokers n (%) n=1,057 (96.8)	Chi square (P Value) for current smokers Vs non-smokers	Prevalence of ever smokers n (%) n=64 (5.9%)	Chi square (P Value) for ever smokers Vs non-smokers
Sex					
Male	25 (5.4)	436 (94.6)		40 (8.7)	
Female	10 (1.6)	621 (98.4)	12.65 (0.000)	24 (3.8)	11.47 (.001)
Age group					
18-24	4 (0.9)	443 (99.1)		12 (2.7)	
25-29	15 (4.2)	345 (95.8)		29 (8.1)	
30-35	16 (5.6)	269 (94.4)	14.09 (0.001)	23 (8.1)	13.84 (.001)
Education level					
None	2 (6.3)	30 (93.8)		4 (12.5)	
Primary	22 (5.1)	409 (94.9)		34 (7.9)	
Post primary	11 (1.8)	618 (98.3)	10.27 (0.006)	26 (4.1)	9.17 (.010)
Occupation					
Agriculture	7 (5.6)	119 (94.4)		8 (6.4)	
Trading	10 (3.1)	308 (96.9)		25 (7.9)	
Housework	5 (2.1)	237 (97.9)		8 (3.3)	
Construction and mechanics workers	6 (5.0)	114 (95.0)		8 (6.7)	
Students	0 (0.0)	50 (98.0)		1 (2.0)	
Boda-boda	2 (3.3)	58 (96.7)		3 (5.0)	
Other	5 (2.9)	170 (97.1)	4.29 (0.508)	11 (6.3)	6.91 (.329)
Marital status					
Married	18 (2.9)	600 (97.1)		29 (4.7)	
Currently not married	7 (4.4)	152 (95.6)		14 (8.8)	
Never been married	10 (3.2)	305 (96.8)	0.91 (0.636)	21 (6.7)	4.40 (.111)
Religion					
None	1 (14.3)	6 (85.7)		1 (14.3)	
Christians	29 (3.7)	746 (96.3)		49 (6.3)	
Muslims	9 (1.9)	304 (98.1)	2.31 (0.128)	14 (4.5)	2.22 (.330)
Alcohol use					
No	7 (0.9)	746 (99.1)		19 (2.5)	
Yes	28 (8.3)	311 (91.7)	40.48 (0.000)	45 (13.3)	48.97 (.000)
HIV-Status					
Negative	27 (2.7)	991 (97.4)		54 (5.3)	
Positive	8 (10.8)	66 (89.2)	14.80 (0.000)	10 (13.5)	8.43 (.004)
Living with friends/relatives who smoke					
No	4 (0.6)	674 (99.4)		10 (1.5)	
Yes	31 (7.5)	383 (92.5)	39.42 (0.000)	54 (13.0)	62.35 (.000)

### Factors Associated With Tobacco Smoking Among Youths Aged 18 to 35 Years

In a bivariate analysis (Table 3), a variety of factors are associated with tobacco smoking for example, older youths (25-29) and (30-35) years were more likely to smoke compared to those aged 18-24 years; (cOR=4.82 [95% CI: 1.58, 14.64], *P*=.006); (cOR=6.59, [95% CI: 2.18, 19.91], *P*=.001), respectively. Other factors that had higher likelihood of smoking included alcohol use (cOR=9.59 [95% CI: 4.15, 22.20], *P*<.0001), HIV positive status (cOR=4.45, [95% CI: 1.95, 10.18], *P*<.001), Living with smokers (cOR=13.64, [95% CI: 4.78, 38.93], *P*<.0001) and including males (cOR=3.56 [95% CI: 1.69, 7.49], *P*=.001). Lastly, marital status, education, religion, and occupation were not associated with tobacco smoking.

**Table 3.** Factors Associated with Tobacco Smoking Among Youths aged (18-35) years, N=1,092

Variable	COR (95%CI) (P Value)	AOR (95%CI) (P Value)
Sex		
Female	1.00	1.00
Male	3.56 (1.69, 7.49) (.001) **	4.50 (1.82, 11.13) (.001) **
Age group		
18-24	1.00	1.00
25-29	4.82 (1.58, 14.64) (.006) **	3.66 (1.15, 11.65) (.028) *
30-35	6.59 (2.18, 19.91) (.001) **	4.26 (1.32, 13.72) (.015) *
Education level		
None	1.00	
Primary	0.81 (0.18, 3.60) (.778)	0.73 (0.14, 3.71) (.702)
Post primary	0.27 (.057, 1.26) (.095)	0.34 (0.06, 1.87) (.215)
Occupation		
Agriculture	1.00	
Trading	0.55 (0.21, 1.48) (.239)	
Housework	0.36 (0.11, 1.15) (.085)	
Construction and mechanics workers	0.89 (0.29, 2.74) (.846)	
Boda-boda	0.59 (0.12, 2.91) (.514)	
Other occupations	0.50 (0.15, 1.61) (.246)	
Marital status		
Married	1.00	
Currently not married	1.54 (0.63, 3.74) (.346)	
Never been married	1.09 (0.50, 2.40) (.825)	
Religion		
Christians	1.00	
Muslims	0.51 (0.21, 1.24) (.135)	
Alcohol use		
No	1.00	1.00
Yes	9.59 (4.15, 22.20) (.000) ***	4.86 (2.01, 11.74) (.000) ***
HIV-Status		
Negative	1.00	1.00
Positive	4.45 (1.95, 10.18) (.000) ***	5.43 (1.86, 15.86) (.002) **
Living with friends/relatives who smoke		
No	1.00	1.00
Yes	13.64 (4.78, 38.93) (.000) ***	9.93 (3.36, 29.29) (.000) ***

*** P<.001

** P<.01

* P<.05

In a multivariable model (Table 3), older youths (25-29) and (30-35) years were more likely to smoke compared to those aged 18-24 years (AOR=3.66, [95% CI: 1.15, 11.65], p=0.028); and (AOR=4.26, [95% CI: 1.32, 13.72], p=0.015) respectively. Other factors associated with higher likelihood of smoking included alcohol use (AOR=4.86, [95% CI: 2.01, 11.74], p<0.0001], HIV-positive status (AOR=5.43, [95% CI: 1.86, 15.86], p=0.002), living with relatives or friends who smoked (AOR=9.93, [95% CI: 3.36, 29.29], p<0.0001) and males (AOR=4.50 [95% CI; 1.82, 11.13], p=0.001) (Table 3).

## DISCUSSION

This study investigated the prevalence of tobacco smoking among youths aged (18-35) years as well as the factors associated with this behavior. These findings show that tobacco-smoking among the youths was still prevalent despite the interventions. Prevalence of tobacco smoking was 3.2% with the age category (30-35) years being dominant. In addition, tobacco smoking was associated with sex, age, HIV positive status, alcohol, living with relatives or friends who smoke.

That the prevalence in this study is 3% and is notable because such a finding is inconsistent with cigarette smoking ranging between 5-10% in Ugandan adolescents. ^28^ The differences in the two studies might have arisen as a result of a comparative study having been conducted among adolescents unlike this research which focused on older youths. This could also partly be explained by numerous efforts by both Ugandan government and private sector in sensitizing masses about the dangers of smoking. Besides that, Ebusu's study was conducted 9 years ago (2014) and therefore may not tell the present time. Besides that, this could as well be explained by the outbreak of COVID-19 pandemic because some studies expressed concern about the more advanced effects of smoking amidst COVID-19 pandemic as this resulted into more deaths among smokers ^29^ therefore this could have reduced the practice of this behavior.

Likewise, other studies from China and Ethiopia indicated that smoking prevalence of 12.5% and 16.9%, respectively.^30,31^ Furthermore, study findings by Tang et al from Kenya and Reddy et al from south Africa revealed higher smoking prevalence of 17% and 17.6%, respectively. ^32,33^ However, the Kenyan study focused on only males aged 15-45 years and this was conducted nine years ago (2014).

Reddy's smoking prevalence was for all persons aged 18-64 years and therefore both studies are not comparable in this study's target population.

Additionally, these population based findings are also different from a study conducted in Tunisia by Zedini et al in 2016 which indicated prevalence of smoking to be high as 22.1%.^34^ we conducted a descriptive cross-sectional study in 2013 to estimate the prevalence of smoking and to identify associated factors among students in Sousse. A questionnaire was administered to a representative sample of 556 students in 5 academic institutions in Sousse randomly drawn. The age of the participants was between 17 and 35 years. The prevalence of tobacco consumption in the past 12 months was 22.1% and consumption during the past 3 months was 65.3%. The average age of starting smoking was 17 years. Smoking prevalence was significantly higher for males (P < 0.001 This study also focused on students in five academic institutions, therefore, may not be representative for illiterate youths aged (18-35) years with a sample of 556 compared to 1,092 participants.

Similar findings by Konfino et al from Argentina indicated smoking prevalence of 3.7%. ^35^ These were similar and this could have arisen from comparative study's higher sample size (2,069). However, although these findings were similar, this study focused on only adolescents aged 13-15 years old compared to 18-35 years old hence not generalizable.

Age was associated with smoking more especially older youths (25+ years) were more likely to smoke compared to younger youths. This finding is consisted with a Ugandan study that found that age is associated with smoking.^36^ The study also found association between alcohol use and tobacco-smoking and this is in line with South African study that focused on tobacco and alcohol use among adolescents (12-17) years.^18^

Furthermore, sex, age, alcohol use, HIV positive youths, living with smokers were the main factors associated with tobacco use and this agrees with findings from a systematic review conducted in Nigeria by Bankole K et al.^37^ Furthermore, similar findings from Saudi Arabia also indicated living with a smoker was associated with smoking. ^38^ Additionally, this study's findings are also boosted by a similar Rwandan study (15-34) years which indicated that older youths were more likely to smoke. ^39^

One of the key finding is that most of the youths (65.71%) want to stop smoking and this is in line with Ghanaian's study which reported (55.6%) hence presenting a fertile ground for future interventions targeted at reducing smoking. ^40^ This study also investigated age at initiation of smoking among youths aged (18-35) years and found out that the average age is 20.6 years. However, this is inconsistent with a US survey that indicated age as 15 years.^11^

### Study Strength

This study has some strengths in that it was conducted by qualified and experienced research assistants from Africa Medical & behavioral Sciences Organization (AMBSO) with high expertise in data collection and probing skills. These are familiar with longitudinal epidemiological research studies and have knowledge on smoking and this significantly improved the quality of the data collected. The sample size of this study was 1,092 and this provided a bigger power of greater than 99%.

### Study Limitations

While this study adopts a longitudinal cohort design, the inherent cross-sectional nature of the data presents challenges in establishing causation. Consequently, we have focused on reporting associations. To gain deeper insights, we advocate for future analytical studies to further explore these relationships. Moreover, it's essential to note that this study relied on secondary data, presenting challenges in the analysis of certain confounding factors that were not originally collected in the primary study.

## CONCLUSIONS

While the prevalence identified in our study is lower than the national average, it remains unacceptably high. To address this concern, targeted initiatives are imperative. Enforcing antismoking campaigns, implementing comprehensive health education programs, and maintaining continuous surveillance are crucial steps. Specifically, efforts should be directed towards males, people living with HIV, alcohol consumers, the aging population, and those residing with relatives who smoke.
